# Supplementary material for: Phytosynthesis of Silver Nanoparticles Using Mansoa alliacea (Lam.) A.H. Gentry (Bignoniaceae) Leaf Extract: Characterization and Their Biological Activities
Source: Pharmaceutics. 2024 Sep 25;16(10):1247. doi: 10.3390/pharmaceutics16101247 (PMC11510252; doi:10.3390/pharmaceutics16101247)
Supplement: Supplementary file 1 [file pharmaceutics-16-01247-s001.zip › pharmaceutics-3182201-supplementary.pdf]

**SUPPLEMENTARY MATERIAL**

# Phytosynthesis of silver nanoparticles using *Mansoa alliacea* (Lam.) A.H. Gentry (Bignoniaceae) leaf extract: characterization and their biological activities.

**Johana Zuñiga-Miranda [1], Saskya E. Carrera-Pacheco [1], Rebeca Gonzalez-Pastor [1], Arianna Mayorga-Ramos [1],**
**Cristina Rodríguez-Pólit [1,], Jorge Heredia-Moya [1], Karla Vizuete [2], Alexis Debut [2,3], Carlos Barba-Ostria [4,5],**
**Elena Coyago-Cruz [6] and Linda P. Guamán [1,*]**

[1] Centro de Investigación Biomédica CENBIO, Facultad de Ciencias de la Salud Eugenio Espejo, Universidad UTE, Quito 170527, Ecuador; johana.zuniga@ute.edu.ec (J.Z.-M.); saskyacarrera@gmail.com (S.E.C.-P.); rebeca.gonzalez@ute.edu.ec (R.G.-P.); arianna.mayorga@ute.edu.ec (A.M.-R.); jorgeh.heredia@ute.edu.ec (J.H.-M.)
[2] Centro de Nanociencia y Nanotecnología, Universidad de Las Fuerzas Armadas ESPE, Sangolquí 171103, Ecuador; ksvizuete@espe.edu.ec (K.V.); apdebut@espe.edu.ec (A.D.)
[3] Departamento de Ciencias de la Vida y Agricultura, Universidad de las Fuerzas Armadas ESPE,
Sangolquí 171103, Ecuador
[4] Escuela de Medicina, Colegio de Ciencias de la Salud Quito, Universidad San Francisco de Quito (USFQ), Quito 170901, Ecuador; cbarbao@usfq.edu.ec
[5] Instituto de Microbiología, Universidad San Francisco de Quito USFQ, Quito 170901, Ecuador
[6] Carrera de Ingeniería en Biotecnología de los Recursos Naturales, Universidad Politécnica Salesiana,
Quito 170143, Ecuador; ecoyagoc@ups.edu.ec

* Correspondence: linda.guaman@ute.edu.ec; Tel.: +593-95-898-6628

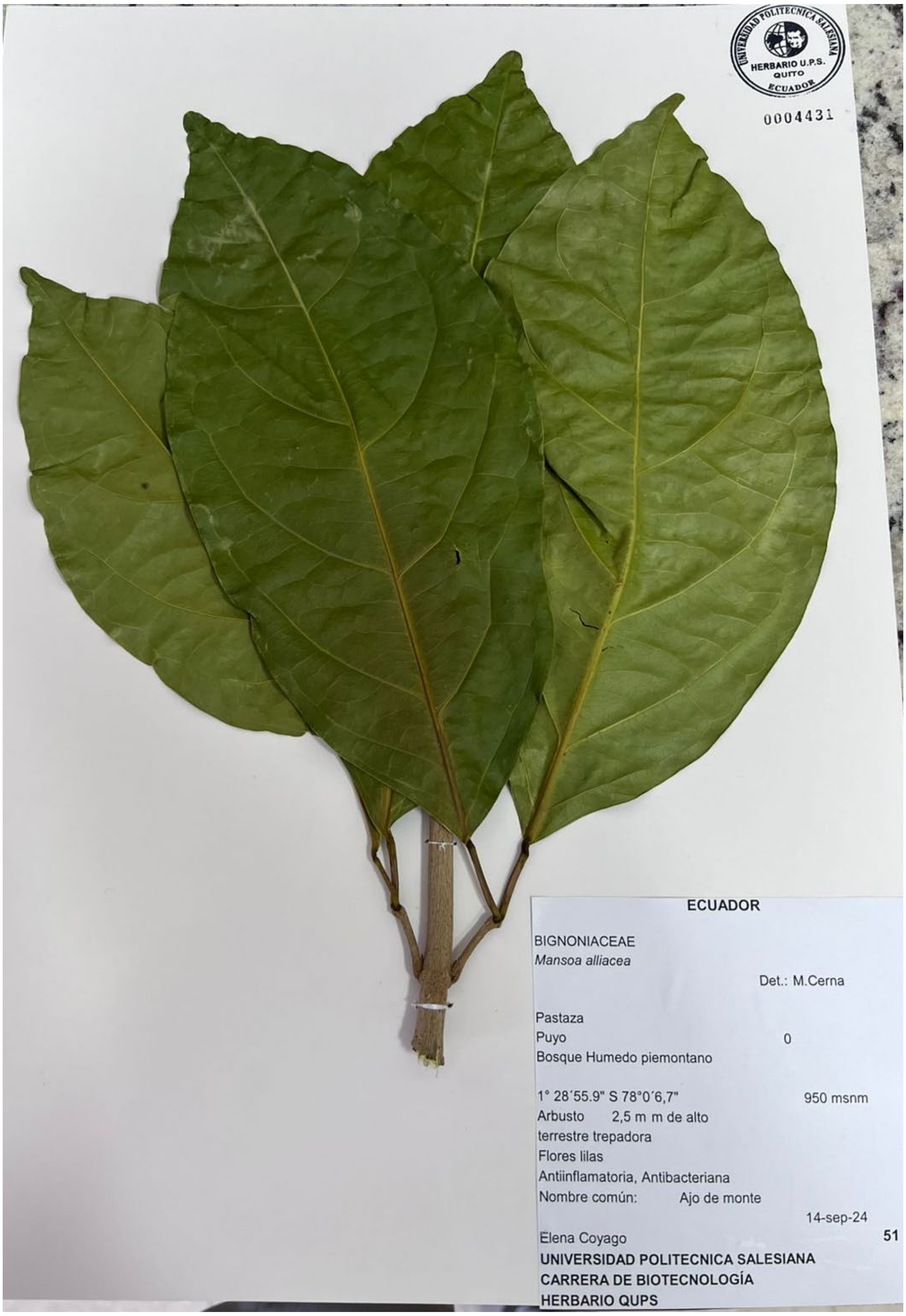


**Figure S1.** Identification of *Mansoa alliacea*.

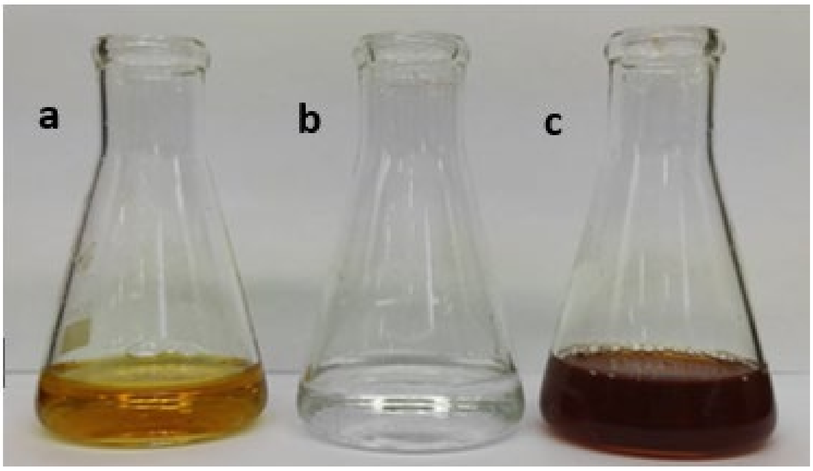


**Figure S2.** Green synthesis of silver nanoparticles, (**a**) leaf extract of *Mansoa alliacea*; (**b**) $AgNO_3$ 10 mM; (**c**) Ma-AgNPs suspension.

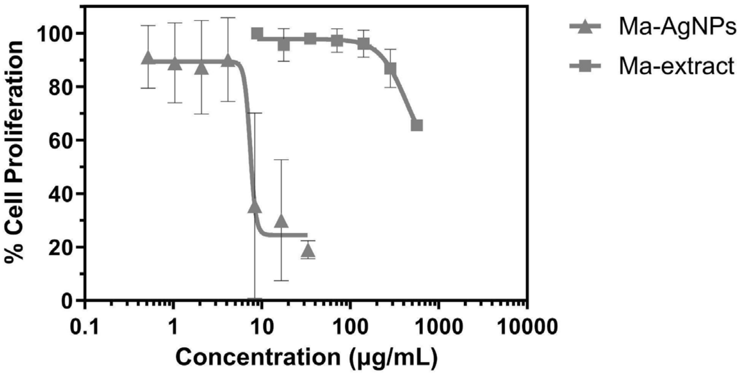


**Figure S3.** Dose-response curves of Ma-AgNPs and Ma-extract against RAW264.7 non-tumor cells. $IC_{20}$ values were calculated to be 7.1 µg/mL for Ma-AgNPs and 371.3 µg/mL Ma-extract.

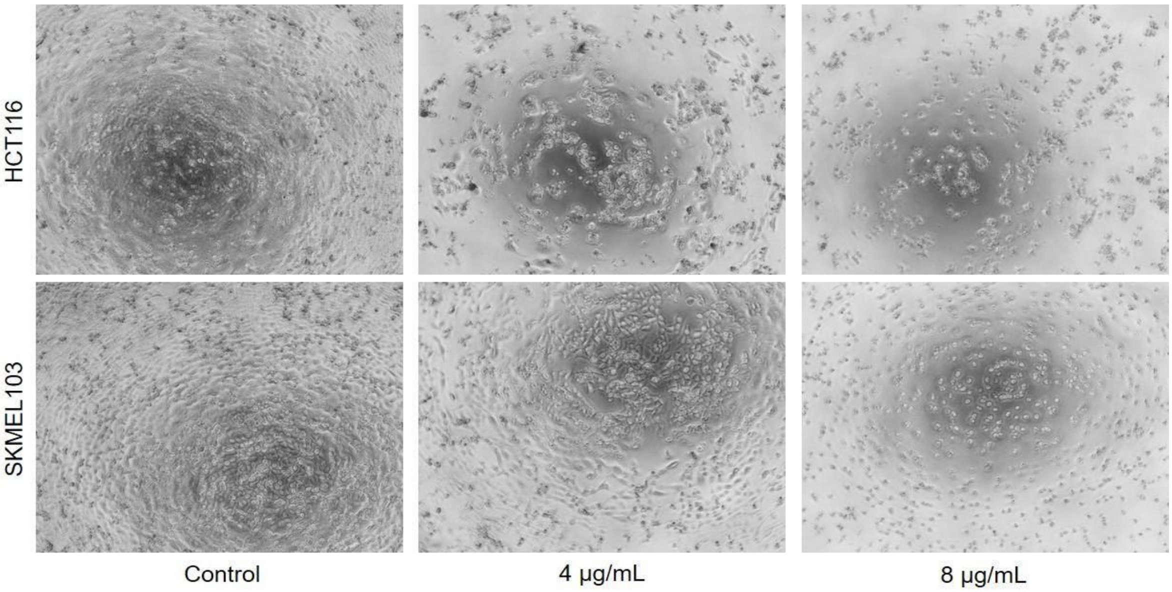

**Figure S4.** Representative images of tumor cells after 72 h of incubation with Ma-AgNPs at two concentrations (4X).

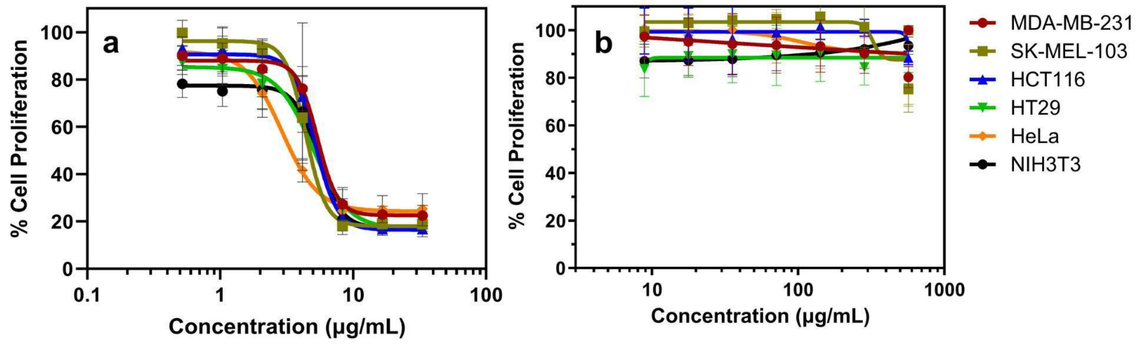


**Figure S5.** Dose-response curves of Ma-AgNPs (left) and Ma-extract (right) against tumor and non-tumor cell lines after 72 h incubation.

**Table S1.** Antibiofilm activity of biosynthesized silver nanoparticles.

| **Microorganism** | **Biofilm Inhibition Assay** | |
|---|---|---|
| | **MBIC50** | **Inhibition rate** |
| *Staphylococcus aureus* **ATCC 25923** | 30 µg/mL ($p<0.05$) | 81 % ± 0.11 |
| *Enterococcus faecalis* **ATCC 29212** | 40 µg/mL ($p<0.05$) | 80 % ± 0.19 |
| *Listeria monocytogenes* **ATCC 13932** | 30 µg/mL ($p<0.05$) | 85 % ± 0.07 |
| *Candida tropicalis* **ATCC 13803** | 40 µg/mL($p<0.05$) | 75 % ± 0.09 |

*MBIC50: Minimum Biofilm Inhibitory Concentration for 50% inhibition

**Table S2.** Antitumor activity of biosynthesized silver nanoparticles and extract. Mean and standard deviation (SD) are presented. This data was used to generate dose-response curves and calculate the $IC_{50}$ values (Supp Material Figures C and D).

**Ma-AgNPs**

| ug/mL | MDA-MB-231 | | SK-MEL-103 | | HCT116 | | HT29 | | HeLa | | NIH3T3 | | RAW264.7 | |
|---|---|---|---|---|---|---|---|---|---|---|---|---|---|---|
| | Mean | SD | Mean | SD | Mean | SD | Mean | SD | Mean | SD | Mean | SD | Mean | SD |
| 0 | 100 | 0 | 100 | 0 | 100 | 0 | 100 | 0 | 100 | 0 | 100 | 0 | 100 | 0 |
| 0.52 | 90.102 | 8.019 | 99.679 | 5.324 | 93.171 | 7.291 | 87.366 | 3.45 | 91.395 | 2.646 | 78.159 | 5.812 | 91.175 | 11.737 |
| 1.04 | 88.877 | 6.509 | 95.267 | 3.082 | 92.677 | 9.621 | 84.435 | 6.17 | 89.645 | 12.212 | 75.06 | 6.446 | 88.973 | 14.967 |
| 2.08 | 84.493 | 8.468 | 92.44 | 3.996 | 84.919 | 12.223 | 78.19 | 10.924 | 74.156 | 10.131 | 75.716 | 7.981 | 87.322 | 17.52 |
| 4.16 | 76.106 | 9.34 | 63.83 | 17.929 | 72.761 | 31.169 | 62.913 | 18.346 | 41.656 | 4.912 | 66.432 | 8.684 | 90.142 | 15.666 |
| 8.33 | 27.301 | 7.071 | 18.285 | 2.323 | 21.517 | 7.098 | 25.641 | 7.92 | 24.471 | 5.272 | 20.813 | 3.504 | 35.508 | 34.688 |
| 16.65 | 22.981 | 3.518 | 18.916 | 3.192 | 16.84 | 2.657 | 18.375 | 2.722 | 25.12 | 5.834 | 20.273 | 3.022 | 29.985 | 22.69 |
| 33.3 | 22.519 | 4.309 | 18.792 | 3.002 | 16.868 | 2.377 | 17.927 | 4.367 | 25.328 | 6.437 | 18.147 | 2.053 | 19.041 | 3.438 |

**Ma-extract**

| ug/mL | MDA-MB-231 | | SK-MEL-103 | | HCT116 | | HT29 | | HeLa | | NIH3T3 | | RAW264.7 | |
|---|---|---|---|---|---|---|---|---|---|---|---|---|---|---|
| 0 | 100 | 0 | 100 | 0 | 100 | 0 | 100 | 0 | 100 | 0 | 100 | 0 | 100 | 0 |
| 8.87 | 97.414 | 9.077 | 99.605 | 6.685 | 99.707 | 9.668 | 83.687 | 11.62 | 100.756 | 12.399 | 87.137 | 7.105 | 100 | 0 |
| 17.73 | 95.204 | 10.015 | 103.021 | 3.401 | 98.166 | 11.221 | 89.163 | 8.876 | 97.732 | 9.282 | 87.328 | 6.345 | 95.627 | 6.184 |
| 35.47 | 94.188 | 12.818 | 104.365 | 2.771 | 96.396 | 14.958 | 89.912 | 12.062 | 100.285 | 10.875 | 87.836 | 7.489 | 98.021 | 0.584 |

| | | | | | | | | | | | | | | |
|---|---|---|---|---|---|---|---|---|---|---|---|---|---|---|
| 70.94 | 93.751 | 11.716 | 104.74 | 4.051 | 99.061 | 5.374 | 89.427 | 12.7 | 96.849 | 10.602 | 89.534 | 6.502 | 97.297 | 4.41 |
| 141.88 | 93.309 | 10.934 | 105.825 | 6.348 | 101.713 | 5.556 | 90.717 | 12.235 | 93.609 | 9.715 | 90.622 | 5.602 | 96.092 | 5.074 |
| 283.75 | 90.289 | 8.397 | 101.439 | 8.692 | 101.063 | 3.583 | 84.52 | 7.497 | 90.727 | 6.196 | 92.079 | 8.203 | 86.884 | 7.201 |
| 567.50 | 80.249 | 4.394 | 75.153 | 9.707 | 88.389 | 2.858 | 75.572 | 6.776 | 81.015 | 13.034 | 93.574 | 3.638 | 65.543 | 1.982 |
